# Office Visitors

**Published:** 1907-07

**Authors:** 


### OFFICE VISITORS.

They were two little sprites. Sisters, but different. Blonde and brunette. One with red hair and freckles, the other with brown hair and fair complexion. Blue and Brown Eyes. One nervous, the other sedate. Brought in by their father and "turned loose" on us to "fix em up." "I'll have the first ride in the doctor's elevator chair," said Blue Eyes, and into it she jumped. "Well if you do I'm to have the doctor tend to me first, so up and down a time or two doctor," demanded Brown Eyes. At the end of the stunt by the doctor there was a squabble as to the possession of the chair but Brown Eyes maintained her serious demand and the chair was relinquished by the other. Brown Eyes stood the ordeal of excavating very well, while Blue Eyes rocked vigorously in the rocking chair awaiting her time. "Does the dental engine hurt," we asked Brown Eyes, "Not much, but it jiggles awful," she answered. She was a good patient and in a short time we finished with her. Then Blue Eyes took a seat, but had to have a ride up and down first before operations began. As we approached her mouth our hand was caught and we were asked, "what are you going to do?" "Fix your teeth." "Well, well, go slow will you and you'll stop when I tell you?" At the first attempt at excavating, the head was jerked violently away and our hand grasped with both of hers. "Why, why, I thought you wasn't to hurt, you were good to Brown Eyes." A good deal of assurance on our part and protests from Brown Eyes enabled us to commence again, when the hand glass had to be produced "to see what tooth you are working on." "Why that ain't the one that hurts, you're fixing the wrong one." Another squabble between the sprites in which Brown Eyes tells Blue Eyes to "shut her mouth," when she wanted the latter to open it and let us proceed. "Wished I didn't have to open it," retorted Blue Eyes. The tooth was ready at last for filling and paper rolls were gotten ready to be put in the mouth. "What are those things—they look like sausages—paper sausages—my!" One was inserted and immediately ejected from the mouth. "Oh! they are horrid, I can't stand 'em—they don't taste like meat ones that's certain." At last it and others go into the mouth of the sprite again and in spite of much wiggling and twisting are kept in position to the end of the operation. "Oh! the nasty things why didn't you use that dam rubber cloth you told us about. Please let me see it. My how it stretches. Say doctor, gimme a strip of it to make a snapper to kill flies with." While the strip was being cut Brown Eyes had forgot her dignity and was experimenting with the engine and a howl from her brought us round and discovered one long curl attached to the bur and spun into coarse twine. "Why the end of it just caught hold of me and before I could stop my foot it had me all wound up." With some difficulty we restored her to herself and then both investigated the cabinet and commented with rapid tongues. We were pleased and annoyed. We were glad they came, we like sprites, and were glad for them to go. We gave them sample tubes of tooth paste which pleased them immensely and they undid them and squirted the stuff on the floor. At last we grasped them both and kissing them both, shoved them through the door and shut it.

